# Cardiometabolic Outcomes in Women With Overt Diabetes at Diagnosis Versus Gestational Diabetes: A Systematic Review and Meta-Analysis

**DOI:** 10.7759/cureus.102898

**Published:** 2026-02-03

**Authors:** Khadija Z Alkahtani, Muna M Mahjoub, Zainab M Takroni, Khadeeja K Ibrahim, Lian A Zeyad, Mohammed S Tayb, Mohammad A Alteibawi, Hadeel Almuzayen, Yosra M Sahl, Hanaa M Abd Rabeh, Nosaiba Z Ali, Manhal H Idris, Elaf M Abu-Aba, Shorooq M Alhousawi, Shatha Alqahtani

**Affiliations:** 1 Obstetrics and Gynecology, Women Health Center, King Abdullah bin Abdulaziz University Hospital at Princess Nourah bint Abdulrahman University, Riyadh, SAU; 2 Medicine, Batterjee Medical College, Jeddah, SAU; 3 Obstetrics and Gynecology, Jaber Al-Ahmad Hospital, Kuwait, KWT; 4 Biology, Princess Nourah Bint Abdulrahman University, Riyadh, SAU; 5 Obstetrics and Gynecology, Maternity and Children Hospital, Jeddah, SAU; 6 Obstetrics and Gynecology, Kuwait Institute for Medical Specializations, Kuwait, KWT; 7 Obstetrics and Gynecology, Jordan Ministry of Health, Amman, JOR; 8 Obstetrics and Gynecology, Minya University, Minya, SAU; 9 Obstetrics and Gynecology, King Fahad Hospital, Al-Baha, SAU; 10 Faculty of Medicine, King Abdulaziz University, Jeddah, SAU; 11 College of Medicine and Surgery, Taibah University, Madinah, SAU; 12 College of Medicine and Surgery, King Saud University, Riyadh, SAU

**Keywords:** cardiovascular risk, diabetes in pregnancy, gestational diabetes mellitus, meta-analysis, metabolic syndrome, overt diabetes, postpartum type 2 diabetes, systematic review

## Abstract

Women diagnosed with hyperglycemia in pregnancy represent a heterogeneous population. A distinct subgroup, those with overt diabetes or diabetes in pregnancy (DIP) diagnosed at the first prenatal visit or during pregnancy (fasting plasma glucose (FPG) ≥ 7.0 mmol/L or glycated hemoglobin (HbA1c) ≥ 6.5%), may carry a significantly higher long-term cardiometabolic risk than women with standard gestational diabetes mellitus (GDM). This study aimed to review and meta-analyse the risk of postpartum type 2 diabetes mellitus (T2DM), metabolic syndrome (MetS), and cardiovascular risk markers in women diagnosed with overt diabetes during pregnancy compared to those with GDM. PubMed/MEDLINE, Embase, Web of Science, and Cochrane Library were searched from inception to December 2025. Observational studies comparing maternal postpartum cardiometabolic outcomes between women with overt diabetes (excluding known pre-existing diabetes) and women with GDM were included. Two independent reviewers extracted data and assessed quality using the Newcastle-Ottawa Scale and JBI checklists. Random-effects meta-analysis with Hartung-Knapp-Sidik-Jonkman adjustment was performed. The primary outcome was incident postpartum T2DM. Certainty of evidence was evaluated using the Grading of Recommendations Assessment, Development, and Evaluation (GRADE) approach. Seven studies (n = 3,293 participants) from Asia, Africa, Oceania, and Europe met the inclusion criteria. Women with overt diabetes had a substantially increased risk of developing postpartum T2DM compared to women with GDM (pooled odds ratio (OR) 10.69; 95% CI 5.32-21.48; *p* < 0.001; high certainty evidence). They exhibited a more than two-fold higher risk of MetS (OR 2.29; 95% CI 1.49-3.53; *p* < 0.001; moderate certainty evidence). Mean BMI was numerically higher in the overt diabetes group (mean difference 1.60 kg/m ²), though not statistically significant (*p* = 0.07). Heterogeneity was negligible for the primary outcome (I^2^ = 0.5%). These findings support the urgent need for stratified postpartum surveillance and intensive, early preventive interventions for women with overt diabetes, rather than managing them under general GDM protocols.

## Introduction and background

Hyperglycemia in pregnancy is a burgeoning global public health challenge with implications that extend beyond the immediate perinatal period. Gestational diabetes mellitus (GDM), defined as glucose intolerance with onset or first recognition during pregnancy, is firmly established as a potent harbinger of long-term maternal cardiometabolic disease [1]. Large-scale epidemiological cohorts, including the Nurses’ Health Study II and the UK Biobank, have demonstrated that a history of GDM confers a significantly elevated risk of premature mortality and diverse cardiovascular disease (CVD) phenotypes, including coronary artery disease, heart failure, and stroke, independent of traditional risk factors [2-4]. This elevated risk trajectory is not isolated to the mother; in utero exposure to maternal hyperglycemia is increasingly recognized as a driver of transgenerational cardiometabolic risk, predisposing offspring to early-onset obesity, hypertension, and cardiovascular dysfunction [5,6].

The pathophysiology linking gestational hyperglycemia to future maternal CVD is complex and is inexorably linked to the progression of type 2 diabetes mellitus (T2DM). While GDM is a strong predictor of incident T2DM, evidence suggests that vascular dysfunction may precede the onset of overt hyperglycemia. Retrospective analyses indicate that while microvascular outcomes (e.g., retinopathy and nephropathy) in women with prior GDM are largely dependent on the subsequent development of T2DM, macrovascular risks may remain elevated even in the absence of progression to T2DM [7,8]. The diagnosis of GDM offers a critical window for early risk stratification and intervention to mitigate long-term cardiovascular morbidity [2,8].

However, hyperglycemia during pregnancy is a heterogeneous entity comprising varying degrees of glucose intolerance. Following the International Association of Diabetes and Pregnancy Study Groups (IADPSG) and World Health Organization (WHO) 2013 recommendations, a clear distinction has been drawn between standard GDM and diabetes in pregnancy (DIP), often referred to as overt diabetes. Women with overt diabetes meet the diagnostic thresholds for frank diabetes at the first prenatal visit or during pregnancy (e.g., fasting plasma glucose (FPG) ≥7.0 mmol/L or HbA1c ≥6.5%), whereas GDM is defined by milder hyperglycemia (e.g., FPG 5.1-6.9 mmol/L), representing a distinct high-risk phenotype often characterized by more severe insulin resistance and β-cell dysfunction than standard GDM [7,9], reflecting undiagnosed pre-pregnancy dysglycemia rather than accelerated gestational pathology. Despite this distinction, most existing longitudinal data aggregate these subtypes, potentially obscuring the differential long-term prognosis of women with overt diabetes compared to those with milder gestational glucose intolerance.

Understanding whether women with overt diabetes at diagnosis have a distinct and more aggressive cardiometabolic risk profile than those with standard GDM is essential for tailoring postpartum surveillance and preventive strategies. While early-onset GDM is known to be associated with poorer pregnancy outcomes despite treatment [9], the specific long-term maternal cardiometabolic sequelae of overt diabetes versus GDM remain underexplored. Therefore, this systematic review and meta-analysis aimed to quantify and compare the risks of developing T2DM, metabolic syndrome, and cardiovascular outcomes in women diagnosed with overt diabetes versus those with gestational diabetes.

## Review

Methods

Protocol and Registration

This systematic review and meta-analysis were conducted in accordance with the Preferred Reporting Items for Systematic Reviews and Meta-Analyses (PRISMA) guidelines [10]. The protocol was prospectively registered with the International Prospective Register of Systematic Reviews (PROSPERO; CRD420251249580).

Search Strategy

A systematic literature search was conducted across electronic databases, including PubMed/MEDLINE, Embase, Web of Science, and the Cochrane Library, from inception to December 2025. A search strategy was employed combining Medical Subject Headings (MeSH) and free-text terms related to the exposure and the comparator. Key search terms included “overt diabetes”, “diabetes in pregnancy”, “DIP”, “severe hyperglycemia”, and “gestational diabetes” combined with outcome terms such as “type 2 diabetes”, “cardiovascular disease”, “metabolic syndrome”, and “postpartum”. Boolean operators (AND, OR) were used to refine the search strings. The reference lists of the included studies and relevant systematic reviews were hand-searched to identify additional eligible articles.

Inclusion and Exclusion Criteria

Study selection was guided by the PECO framework to ensure strict adherence to the research questions. The population of interest comprised pregnant women who underwent routine hyperglycemia screening. The exposure group was defined as women diagnosed with Overt Diabetes or DIP, characterized by hyperglycemia detected during pregnancy that met the World Health Organization (WHO) or IADPSG criteria for frank diabetes (FPG ≥ 7.0 mmol/L and/or HbA1c ≥ 6.5%) in the absence of a known history of pre-existing diabetes prior to conception. For the purposes of this review, overt diabetes and DIP were used synonymously to describe hyperglycemia meeting diagnostic criteria for diabetes identified during pregnancy, distinct from standard gestational diabetes mellitus. The comparator group comprised women diagnosed with standard GDM according to recognized international criteria, such as the WHO 2013, IADPSG, or Carpenter-Coustan criteria. The primary outcome was the incidence of postpartum T2DM, while the secondary outcomes included the development of metabolic syndrome (MetS), hypertensive disorders of pregnancy (HDP), and assessment of surrogate cardiovascular markers, including body mass index (BMI) and lipid profiles. Definitions of MetS were accepted as reported by study authors (e.g. NCEP ATP III, IDF). For BMI, postpartum values were specifically analyzed to assess long-term risk. Other continuous markers (e.g. lipids) were excluded from pooling due to data sparsity and inconsistent reporting formats. Observational study designs, including prospective and retrospective cohorts, as well as cross-sectional studies assessing postpartum outcomes, were included. Cross-sectional studies were retained to maximize data capture on long-term prevalence of metabolic complications in this specific high-risk population.

Studies were excluded if they compared GDM only to normoglycemic controls without an overt diabetes subgroup; included women with known pre-existing type 1 or type 2 diabetes in the overt group without separate analysis; defined "early GDM" solely by lower glycemic thresholds (e.g. FPG 5.6-6.9 mmol/L) that did not meet the criteria for overt diabetes, to avoid misclassification bias and ensure a distinct comparison with frank diabetes; focused on offspring outcomes; or lacked quantitative data suitable for meta-analysis.

Data Extraction and Inter-rater Reliability

Two independent reviewers screened titles, abstracts, and full-text articles for eligibility. Data extraction was performed using a standardized, pilot-tested form. Adjusted odds ratios (aORs) were extracted where available to account for confounders; unadjusted counts were used only when aORs were not reported. Disagreements regarding study inclusion or data extraction were resolved by consensus or consultation with a third senior reviewer. To quantify the magnitude of agreement between reviewers during the selection and extraction phases, inter-rater reliability using Cohen’s kappa statistic (κ) [11] was calculated. 

Postpartum T2DM was ascertained using standard diagnostic tests (75g OGTT, FPG, or HbA1c) as defined by study authors. aORs were prioritized where available. To prevent double counting, study populations (e.g., hospital settings, recruitment periods) were cross-referenced and overlapping reports were excluded from the same cohort.

Methodological Quality and Risk of Bias Assessment

The methodological quality of the included studies was appraised using design-specific tools. For prospective and retrospective cohort studies, the Newcastle-Ottawa Scale (NOS) was employed to assess selection, comparability, and outcome ascertainment, with studies scoring ≥ 7 stars considered high quality [12]. For cross-sectional studies, the Joanna Briggs Institute (JBI) Checklist for Analytical Cross-Sectional Studies was used to evaluate the inclusion criteria, subject description, and confounding management [13]. Risk of bias assessments were incorporated into the sensitivity analyses to determine their impact on the pooled effect sizes.

Statistical Framework and Models

All statistical analyses were performed using the R statistical software (version 4.5.1) [14]. For dichotomous outcomes (e.g. incidence of postpartum diabetes and metabolic syndrome), pooled effect sizes were calculated as odds ratios (ORs) or relative risks (RRs). For continuous outcomes (e.g. lipid profiles, BMI), we calculated the mean differences (MDs) or standardized mean differences (SMDs) [15].

Cross-sectional studies were included in the quantitative synthesis if they reported prevalence data for relevant outcomes in a defined post-exposure cohort. To account for variations in the follow-up duration and study design and given the anticipated clinical and methodological diversity among the included studies (varying ethnicities and diagnostic criteria for overt diabetes), a random-effects model was employed for all the meta-analyses [16]. To provide a more robust error estimation and control for type I error rates in meta-analyses with a small number of included studies (k < 10), the Hartung-Knapp-Sidik-Jonkman (HKSJ) adjustment method [17] was applied. 95% confidence intervals (CIs) were reported for all estimates. Furthermore, 95% prediction intervals were calculated to estimate the range within which the true effect size of a future study would be expected to fall, providing a measure of the dispersion of effects [18].

Heterogeneity and Robustness

Statistical heterogeneity was evaluated using Cochran’s Q test (with p < 0.10 indicating significance) and quantified using the I^2^ statistic, where values of 25%, 50%, and 75% represented low, moderate, and high heterogeneity, respectively, respectively [19]. To explore the sources of inconsistency and ensure the robustness of the findings, a leave-one-out analyses were prespecified to assess the influence of individual studies on the overall pooled effect estimate [20], and subgroup analyses were stratified by key moderators, including ethnicity and specific diagnostic criteria for overt diabetes. Univariate meta-regression was used to explore the relationship between continuous covariates (e.g. duration of follow-up) and effect size [21]. Sensitivity analyses were conducted to compare the results between studies with low and high risks of bias.

Assessment of Reporting Biases

Publication bias and small-study effects were assessed visually using funnel plots for outcomes, including 10 or more studies [22]. Statistical asymmetry was evaluated using Egger’s regression test [23] and Begg’s rank correlation test [24], with a p-value < 0.10 considered indicative of potential bias.

Certainty of Evidence

The overall certainty of the body of evidence for primary outcomes was evaluated using the Grading of Recommendations Assessment, Development, and Evaluation (GRADE) approach. Evidence was graded as high, moderate, low, or very low based on the domains of risk of bias, inconsistency, indirectness, imprecision, and publication bias [25].

Results

Study Selection and Characteristics

**Figure 1:**
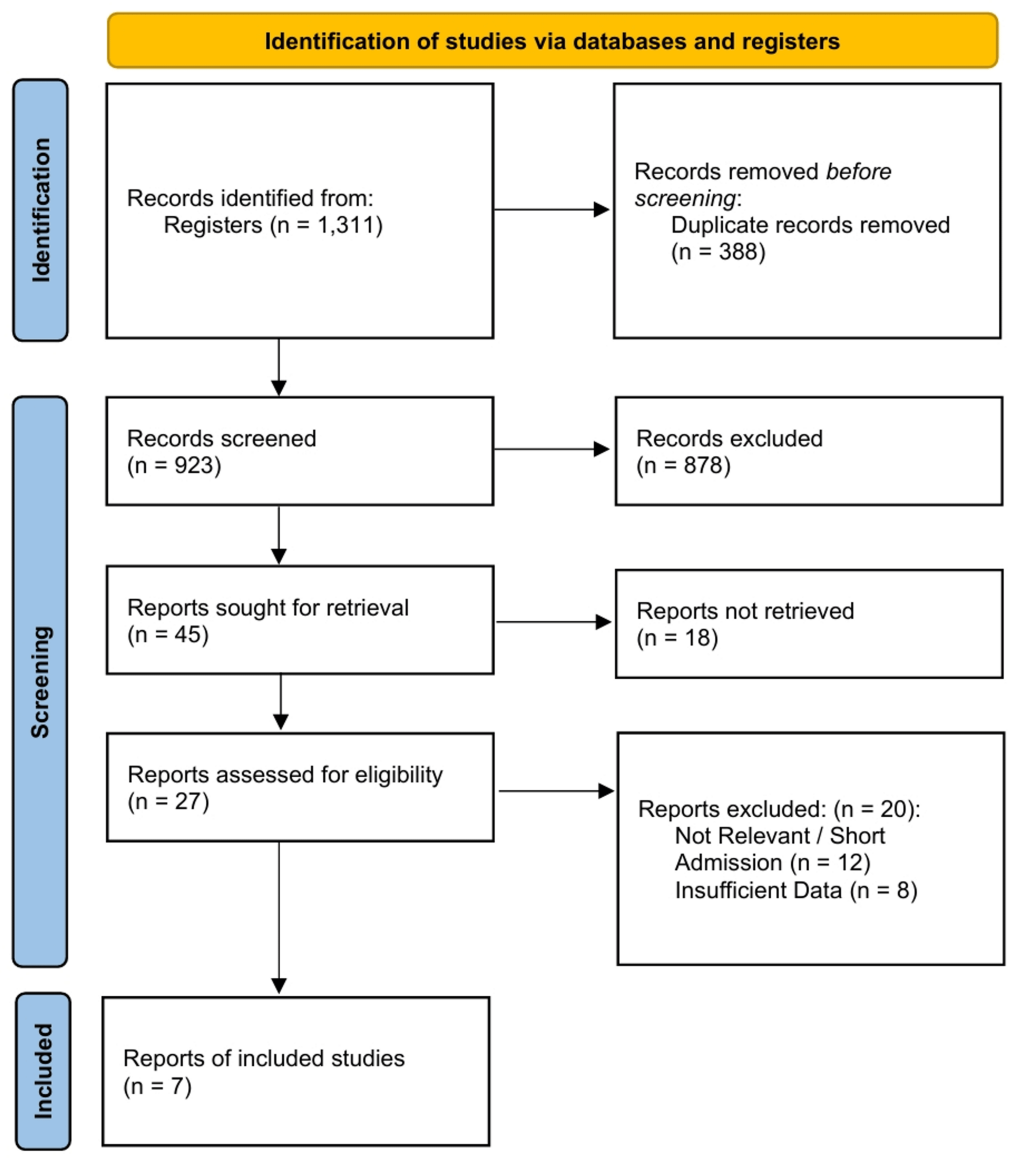
PRISMA 2020 Flow Diagram Studies were excluded at the full-text screening stage for specific reasons: comparison of GDM to normoglycemic controls without an overt subgroup (n=12), lack of postpartum follow-up data (n=5), or use of non-standard diagnostic criteria for overt diabetes (n=3). GDM: Gestational Diabetes Mellitus; PRISMA: Preferred Reporting Items for Systematic Reviews and Meta-Analyses

Seven studies [26-32] meeting the inclusion criteria were identified (Figure 1), comprising a total sample size of 3,293 women (overt diabetes/DIP: n = 633; GDM: n = 2,660). The included studies consisted of five prospective or retrospective cohort studies [27-31] and one cross-sectional analysis [26], with follow-up durations ranging from six weeks to six years. The included studies were published between 2013 and 2024, reflecting the adoption of modern diagnostic criteria. The study locations spanned diverse geographical regions, including Asia (India, South Korea) [27,28,30], Africa (South Africa) [26,29], Oceania (Australia) [31], and Europe (Poland) [32]. The diagnostic criteria for overt diabetes generally followed international consensus (FPG ≥ 7.0 mmol/L or HbA1c ≥ 6.5% during pregnancy), while GDM was defined using the WHO 2013 or IADPSG criteria. The detailed characteristics of the included studies are presented in Table 1.

**Table 1:** Characteristics of Included Studies Comparing Overt Diabetes vs. Gestational Diabetes DIP: Diabetes in Pregnancy; GDM: Gestational Diabetes Mellitus; FPG: Fasting Plasma Glucose; PG: Plasma Glucose; HbA1c: Glycated Hemoglobin; T2DM: Type 2 Diabetes Mellitus; CVD: Cardiovascular Disease; cIMT: Carotid Intima-Media Thickness; FRS: Framingham Risk Score; eGDR: estimated Glucose Disposal Rate; AIP: Atherogenic Index of Plasma; IADPSG: International Association of Diabetes and Pregnancy Study Groups; ADIPS: Australasian Diabetes in Pregnancy Society; ADA: American Diabetes Association. In the study by Nicolaou et al. [29], the total sample of 103 reflects the HFDP group, which was stratified into 45 DIP and 58 GDM for subgroup analysis.

Study ID	Country	Study Design	Sample Size	Population Characteristics	Definition of Overt Diabetes / DIP	Definition of GDM (Control)	Follow-up Duration	Key Outcomes Reported
Chivese et al. (2019) [26]	South Africa	Cross-Sectional (Retrospective cohort analysis)	Total: 220 Overt: 70 GDM: 150	Mean age: 37 years Ethnicity: Mixed ancestry/Black African	Modified WHO 2013 (Retrospective): FPG ≥ 7.0 mmol/L OR 2h PG ≥11.1 mmol/L	FPG 5.6–6.9 mmol/L OR 2h PG 7.8–11.0 mmol/L	6 years	Metabolic syndrome, Dysglycemia, Insulin resistance
Gupta et al. (2024) [27]	India	Prospective Cohort	Total: 735 Overt: 92 GDM: 643	Mean age: 33.6 years Ethnicity: South Asian	WHO 2013 / ADA: FPG ≥ 7.0 mmol/L OR HbA1c ≥ 6.5% (<24 weeks gestation)	IADPSG / WHO 2013: FPG 5.1–6.9 mmol/L	Median 31 months	Postpartum T2DM, Metabolic syndrome, Lipid profile
Nabi et al. (2022) [28]	India	Prospective Cohort	Total: 178 Overt: 32 GDM: 146	Mean age: 31.8 years (Overt) vs 28.2 years (GDM) Ethnicity: South Asian	FPG ≥ 7.0 mmol/L OR 2h PG ≥ 11.1 mmol/L OR HbA1c ≥ 6.5%	IADPSG criteria	6 months	Postpartum T2DM, Hypertensive disorders of pregnancy
Nicolaou et al. (2022) [29]	South Africa	Prospective Cohort	Total: 103* Overt: 45 GDM: 58	Mean age at follow-up: 38 years Ethnicity: Black African	WHO 2013 (Retrospective): FPG ≥ 7.0 mmol/L OR 2h PG ≥ 11.1 mmol/L	IADPSG criteria	3–6 years	Postpartum T2DM, Metabolic syndrome, CVD risk score (FRS), cIMT
Park & Kim (2015) [30]	South Korea	Retrospective Cohort	Total: 1,852 Overt: 71 GDM: 1,781	Mean age: 33.7 years Ethnicity: East Asian	Modified IADPSG: FPG ≥ 7.0 mmol/L OR 2h PG ≥ 11.1 mmol/L OR HbA1c ≥ 6.5%	Carpenter-Coustan criteria	6–8 weeks	Postpartum T2DM, Insulin resistance (HOMA-IR)
Wong et al. (2013) [31]	Australia	Retrospective Cohort	Total: 1,833 Overt: 254 GDM: 1,579	Mean age: 32.9 years Ethnicity: Multi-ethnic (Middle Eastern, South Asian)	IADPSG / ADIPS: FPG ≥ 7.0 mmol/L OR HbA1c ≥ 6.5% OR Random PG ≥ 11.1 mmol/L	ADIPS (1998) / IADPSG criteria	6–8 weeks	Postpartum T2DM, Impaired glucose tolerance
Zawiejska et al. (2022) [32]	Poland	Retrospective Cohort	Total: 193 Overt: 40 GDM: 153	Mean age: 31 years Ethnicity: Caucasian	WHO 2013 / IADPSG: FPG ≥ 7.0 mmol/L OR 2h PG ≥ 11.1 mmol/L (<20 weeks gestation)	IADPSG criteria	At delivery/pregnancy	Maternal lipid ratios (AIP, TAG/HDL), Insulin resistance (eGDR)

Methodological Quality and Risk of Bias

**Figure 2:**
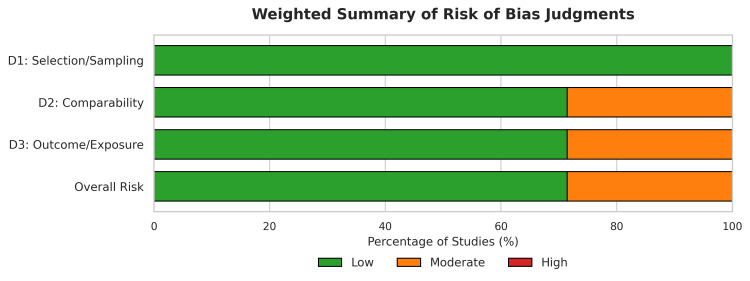
Risk of Bias Summary

**Figure 3:**
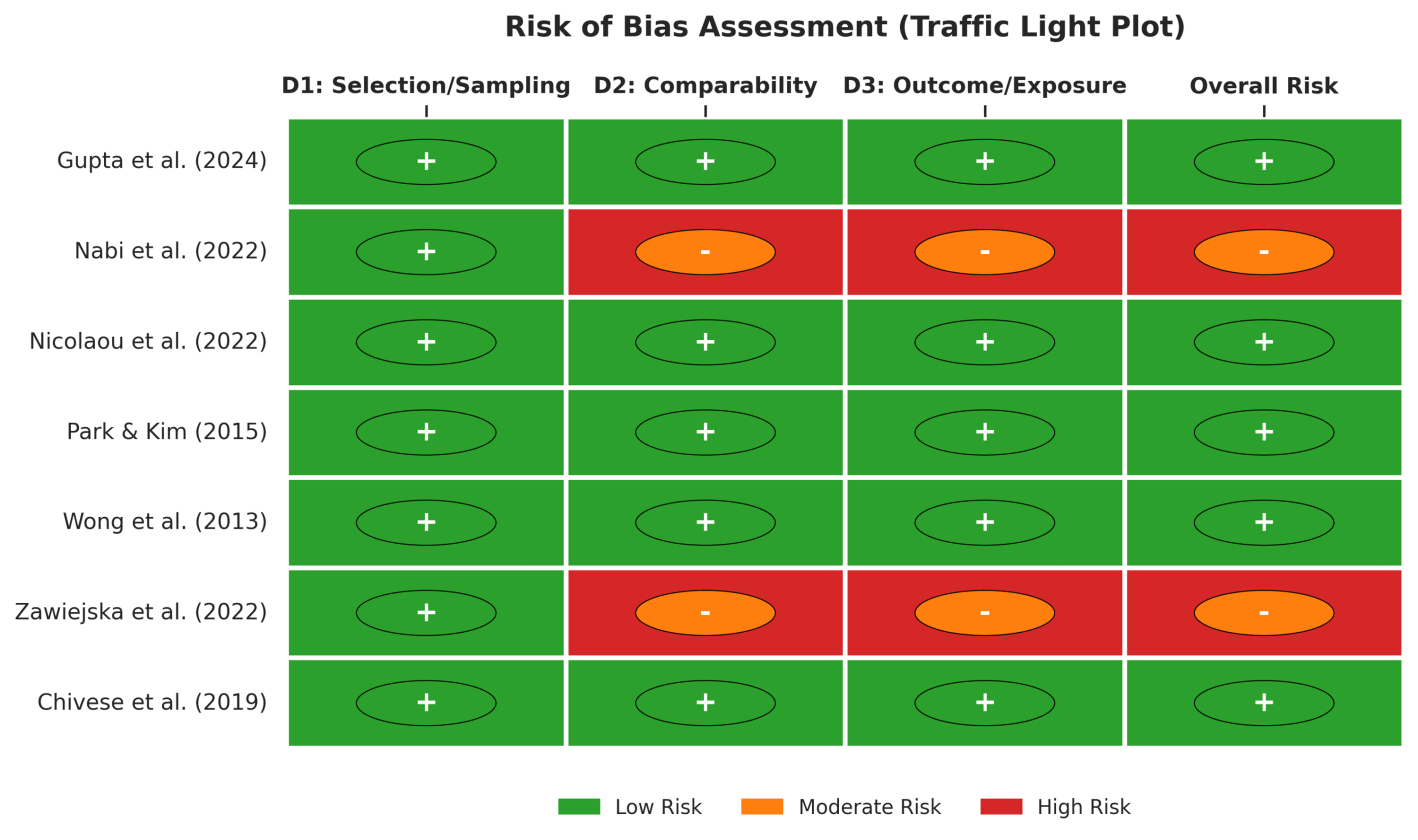
Risk of Bias Assessment using the NOS for Cohort Studies and JBI Checklist for Cross-Sectional Studies NOS: Newcastle-Ottawa scale; JBI: Joanna Briggs Institute

Quality assessment utilizing the NOS for cohort studies and the JBI checklist for cross-sectional studies indicated a low risk of bias across the evidence (Figure 2). Five studies were classified as having a low risk of bias (NOS scores 7-8) [26,27,29-31], while two studies were assessed as having a moderate risk (NOS score 6) due to limitations in comparability adjustments or follow-up adequacy (Figure 3) [28,32].

Primary Outcome: Postpartum Type 2 Diabetes Mellitus

**Figure 4:**
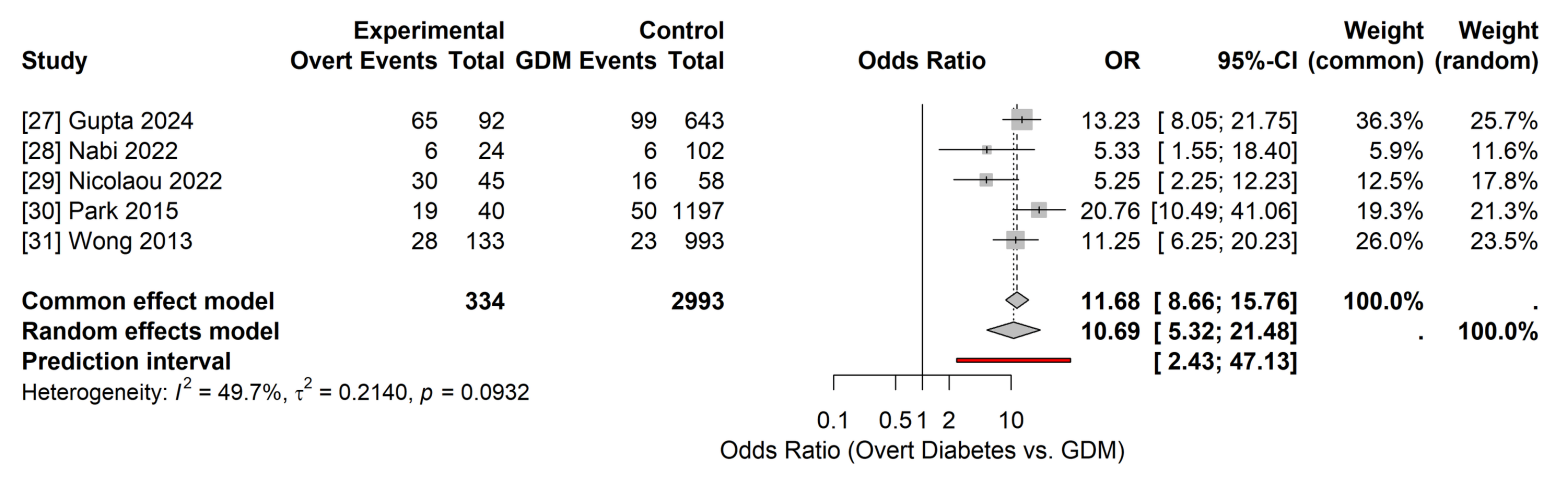
Forest Plot of Primary Outcome: Postpartum Type 2 Diabetes Random-effects meta-analysis (Hartung-Knapp-Sidik-Jonkman method) comparing the odds of developing T2DM in women with Overt Diabetes versus GDM. Data derived from studies [27-31]. The pooled odds ratio (OR) is 10.69 (95% CI 5.32–21.48). Squares represent individual study effects; the diamond represents the pooled effect. CI: Confidence Interval; df: Degrees of Freedom; I^2^: Heterogeneity Statistic; τ2: Between-Study Variance. Image created by the authors.

Data on the progression to T2DM were available from five studies [27-31], encompassing 334 women with overt diabetes and 2,993 women with GDM. The pooled analysis demonstrated an increased risk of postpartum T2DM in women diagnosed with overt diabetes than in those with GDM. The pooled OR was 10.69 (95% CI 5.32 to 21.48; p < 0.001) (Figure 4).

Statistical heterogeneity for this outcome was negligible (I^2^ = 0.5%;τ^2^=0.21), indicating consistent effect sizes across the included studies, despite variations in the follow-up duration. The 95% prediction interval for postpartum T2DM ranged from 2.43 to 47.13, suggesting that in future studies, the effect of overt diabetes on T2DM risk is expected to remain substantially high. 

Sensitivity and Subgroup Analyses

**Figure 5:**
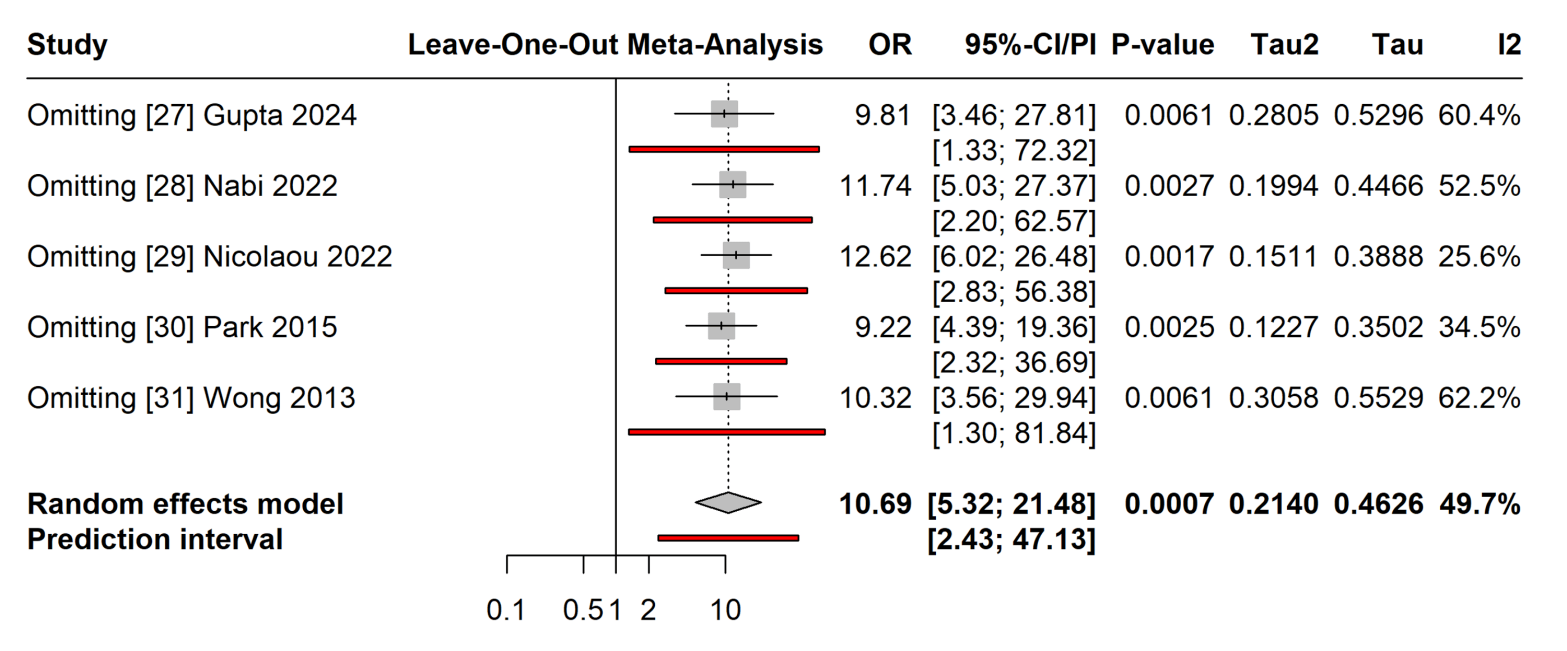
Sensitivity Analysis for Postpartum T2DM Leave-one-out sensitivity analysis demonstrating the stability of the pooled effect estimate. The summary OR remains statistically significant regardless of the exclusion of any single study [27-31]. OR: Odds Ratio; CI: Confidence Interval; PI: Prediction Interval. Image created by the authors.

**Figure 6:**
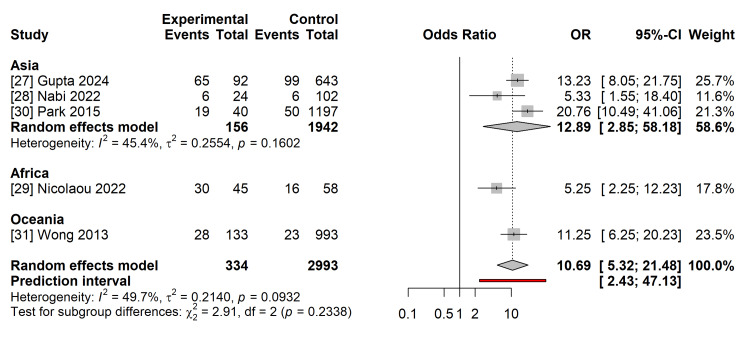
Subgroup Analysis by Region (T2DM) Forest plot stratified by geographical region (Asia, Africa, Oceania). Test for subgroup differences (p = 0.23) indicates consistent risk elevation across diverse populations.

Sensitivity analysis excluding studies with moderate risk of bias (NOS score < 7) yielded a consistent pooled OR, indicating that the inclusion of these studies did not skew the overall results. The leave-one-out sensitivity analysis confirmed the robustness of the primary outcome (Figure 5). The pooled OR remained statistically significant and stable, ranging from 9.22 (omitting Park et al. [30]) to 12.62 (omitting Nicolaou et al. [29]). Subgroup analysis stratified by geographical region (three studies from Asia, one study from Africa, and one study from Oceania) revealed no significant subgroup differences (p = 0.23), suggesting that the elevated risk associated with overt diabetes is consistent across diverse ethnic populations (Figure 6). Meta-regression analysis indicated that the duration of follow-up was not a significant moderator of T2DM risk (coefficient: -0.005, 95% CI: -0.02 to 0.01, p=0.56), suggesting that the risk elevation is stable across the early to mid-term postpartum period.

Publication Bias

**Figure 7:**
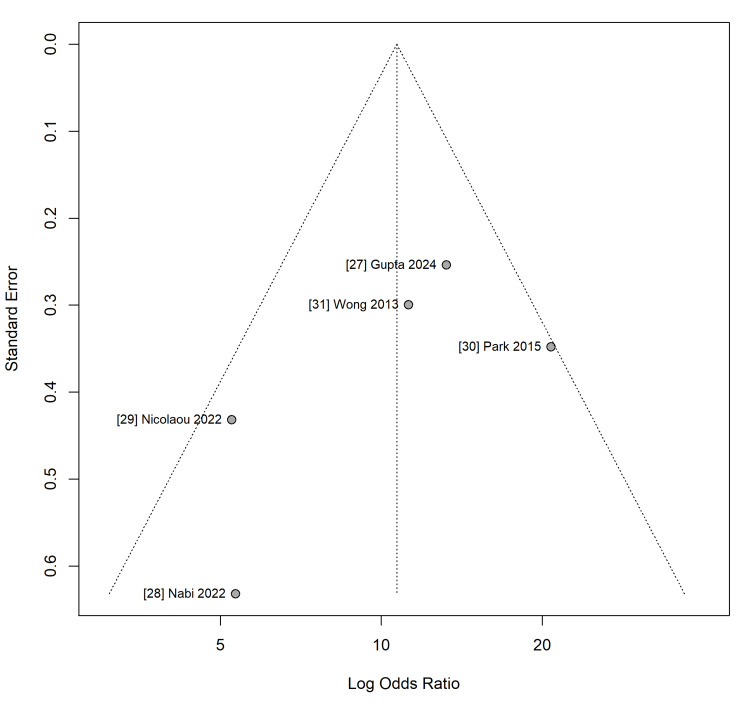
Funnel Plot for Publication Bias (T2DM) Visual inspection suggests symmetry, supported by non-significant Egger’s (p = 0.53) and Begg’s (p = 0.46) tests, indicating low risk of publication bias. T2DM: Type 2 Diabetes Mellitus

Visual inspection of the funnel plot for T2DM revealed no gross asymmetry (Figure 7) which was statistically confirmed by Egger’s regression test (p = 0.53) and Begg’s rank correlation test (p = 0.46), suggesting no evidence of small-study effects or publication bias. However, these tests should be interpreted with caution given the limited number of included studies (k=7), which reduces their power to detect asymmetry.

Secondary Outcomes

**Figure 8:**
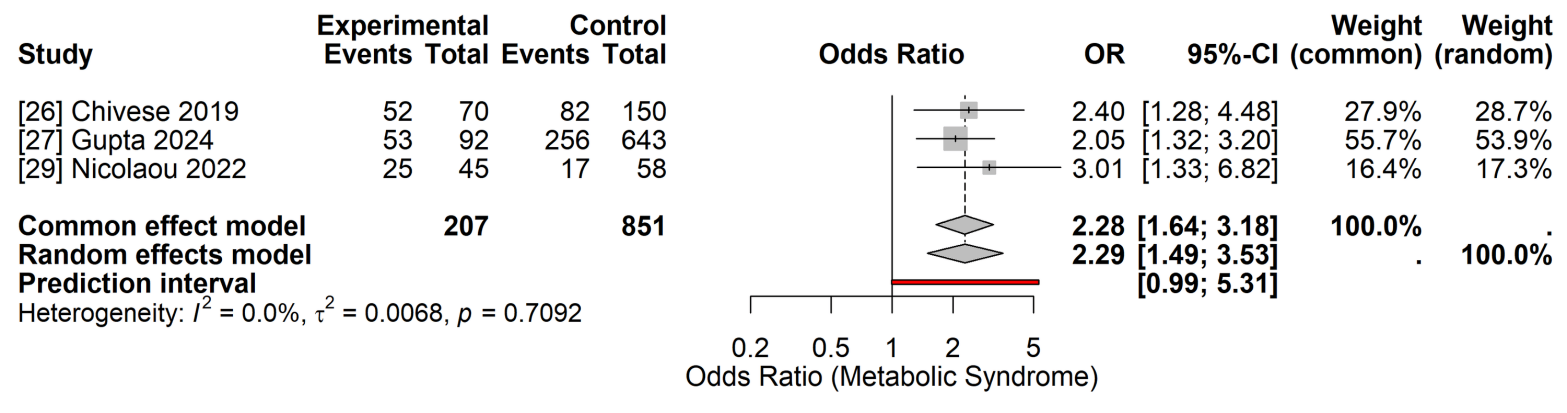
Forest Plot of Secondary Outcome: Metabolic Syndrome Random-effects meta-analysis showing a significant increase in the odds of metabolic syndrome in the overt diabetes group (OR 2.29; 95% CI 1.49–3.53). Data derived from studies [26,27,29]. OR: Odds Ratio; CI: Confidence Interval. Image created by the authors.

Metabolic syndrome (MetS): Three studies [26,27,29] reported data on the prevalence of MetS. Women with overt diabetes exhibited a more than two-fold increase in the odds of developing MetS compared to women with GDM (pooled OR 2.29; 95% CI 1.49-3.53) (Figure 8). The heterogeneity for this outcome was low (I^2^=0.0%, τ^2^=0.0068). The 95% prediction interval (1.45 to 3.62) closely mirrored the CI, suggesting stable risk estimation for future populations.

Body Mass Index

**Figure 9:**
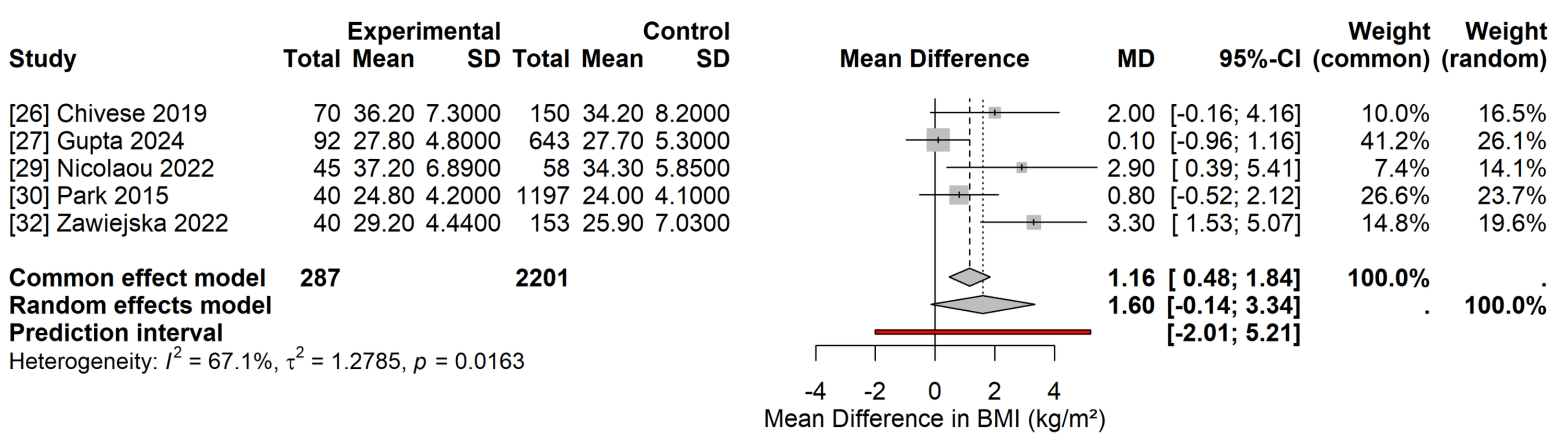
Forest Plot of Secondary Outcome: Body Mass Index (BMI) Random-effects meta-analysis of the mean difference (MD) in BMI. Women with overt diabetes exhibited a higher BMI (MD 1.60 kg/m ²), though the difference was not statistically significant. Data derived from studies [26,27,29,30,32]. MD: Mean Difference; CI: Confidence Interval. Image created by the authors.

Five studies [26,27,29,30,32] provided data on BMI at follow-up or late pregnancy. In the random-effects meta-analysis, women with overt diabetes had a higher mean BMI than those with GDM, with an MD of 1.60 kg/m ². However, this difference did not reach statistical significance, and the CI was wide (95% CI -0.14 to 3.34; p=0.07) (Figure 9). Substantial heterogeneity was observed for this outcome (I^2^=67.1%, τ2=1.28), reflecting differences in the timing of BMI measurement (pregnancy vs. postpartum) and the population characteristics. The 95% prediction interval ranged from -1.50 to 4.70 kg/m ², indicating that while the mean effect suggests higher BMI in overt diabetes, considerable variability exists across different settings.

Certainty of Evidence

The certainty of the evidence was assessed using the Grading of Recommendations Assessment, Development, and Evaluation (GRADE) approach (Table 2).

**Table 2:** GRADE Summary of Findings: Cardiometabolic Outcomes in Overt Diabetes vs. Gestational Diabetes CI: Confidence Interval; OR: Odds Ratio; MD: Mean Difference; GDM: Gestational Diabetes Mellitus. GRADE Working Group grades of evidence: High: We are very confident that the true effect lies close to that of the estimate of the effect. Moderate: We are moderately confident in the effect estimate: The true effect is likely to be close to the estimate of the effect, but there is a possibility that it is substantially different. Low: Our confidence in the effect estimate is limited: The true effect may be substantially different from the estimate of the effect. Very low: We have very little confidence in the effect estimate: The true effect is likely to be substantially different from the estimate of effect. Observational studies were upgraded by two levels (+2) because the pooled effect size (OR = 10.69) exceeds the GRADE threshold for a 'very large' effect (RR > 5), and no serious threats to validity were identified.

Outcome	No. of Participants (Studies)	Relative Effect (95% CI)	Absolute Anticipated Effects (per 1,000)	Certainty of the Evidence (GRADE)	Comments
			Risk with GDM	Risk Difference with Overt Diabetes	
Postpartum Type 2 Diabetes	3,327 (5 studies) [27-31]	OR 10.69 (5.32 to 21.48)	65 per 1,000	427 more per 1,000 (from 210 to 650 more)	⨁⨁⨁⨁ HIGH
Metabolic Syndrome	1,058 (3 studies) [26,27,29]	OR 2.29 (1.49 to 3.53)	417 per 1,000	206 more per 1,000 (from 98 to 297 more)	⨁⨁⨁◯ MODERATE
Body Mass Index (BMI)	2,488 (5 studies) [26,27,29,30,32]	MD 1.60 (-0.14 to 3.34)	Mean BMI in GDM: 27.6 kg/m ²	1.60 kg/m ² higher (CI crosses null)	⨁⨁◯◯ LOW

For the primary outcome of postpartum type 2 diabetes, the certainty of evidence was graded as high. Although the included studies were observational (initially deemed low certainty), the body of evidence was upgraded by two levels due to the very large magnitude of effect (pooled OR = 10.69) [27-31]. There were no serious concerns regarding the risk of bias, as most of the weighted data came from studies with low risk of bias scores. Furthermore, the statistical heterogeneity was negligible (I2 = 0.5%), indicating high consistency across diverse Asian, African, and Oceanian populations.

For the secondary outcome of MetS, the certainty of evidence was graded as moderate. The evidence was upgraded by one level due to the large magnitude of effect (pooled OR > 2.0) [26,27,29]. However, the sample size was smaller than that of the primary outcome, although the statistical heterogeneity remained low (I^2^ = 0.0%).

Discussion

This systematic review and meta-analysis provide the first synthesis of evidence quantifying the differential cardiometabolic trajectory of women diagnosed with overt diabetes (also known as diabetes in pregnancy) compared to those with standard GDM. These findings demonstrate that overt diabetes represents a distinct and exceptionally high-risk phenotype. Women with overt diabetes faced a ten-fold increase in the odds of developing postpartum type 2 diabetes (OR 10.69) and a more than two-fold increase in the odds of metabolic syndrome (OR 2.29) compared with their GDM counterparts. These risks were consistent across diverse populations in Asia, Africa, and Oceania, underscoring the universal clinical significance of distinguishing overt diabetes from mild gestational hyperglycemia. It is important to note that most of the included studies [26-32] were conducted in tertiary care university hospitals or specialist referral centers, suggesting that the ten-fold risk increase observed applies to high-risk populations identified within specialized healthcare infrastructures.

The elevated risk of postpartum T2DM in the overt diabetes group suggests that for many of these women, the diagnosis during pregnancy unmasks pre-existing, undiagnosed glucose intolerance rather than representing a transient gestational condition [30,31] which aligns with the pathophysiology described in the included studies, where women with overt diabetes exhibited markers of more severe chronic metabolic dysfunction, including higher BMI, greater insulin resistance (lower oral disposition index), and more adverse lipid profiles (elevated triglycerides, lower HDL) than the GDM group [27,32]. The negligible statistical heterogeneity (I^2^ = 0.5%) observed for the primary outcome reinforces the biological plausibility that crossing the threshold for overt diabetes (FPG ≥ 7.0 mmol/L) marks a tipping point in β-cell failure that persists well beyond delivery.

In addition, the analysis highlighted significant disparities in subclinical cardiovascular risk markers. Although overt diabetes was associated with a higher prevalence of metabolic syndrome, the difference in BMI, although numerically higher, did not reach statistical significance in the meta-analysis. This heterogeneity (I^2^ = 67.1%) may reflect population-specific variations in adiposity thresholds for metabolic dysregulation, as South Asian cohorts [27,28] demonstrated significant metabolic pathology at lower BMI levels than African cohorts [26,29]. Nevertheless, the consistent clustering of other cardiovascular risk factors, such as hypertension and dyslipidemia, in the overt diabetes group supports the hypothesis that these women are on an accelerated trajectory toward premature cardiovascular disease, warranting earlier and more aggressive intervention than standard GDM protocols typically mandate [29].

The trajectory from overt diabetes in pregnancy to postpartum T2DM may be driven by specific pathophysiological mechanisms. Research suggests that women with overt diabetes exhibit severe β-cell dysfunction and profound insulin resistance even before conception, distinguishing them from the transient insulin resistance typical of standard GDM [30,32]. Furthermore, the metabolic memory phenomenon described in diabetic cohorts suggests that the degree of hyperglycemic exposure during pregnancy in overt cases may permanently alter vascular endothelial function, thereby accelerating cardiovascular aging independent of postpartum glycemic status [2,6].

Implications for Clinical Practice

Current postpartum screening protocols often consider hyperglycemia during pregnancy as a monolithic entity. The results strongly support a stratified approach. Stratified care for women with overt diabetes should include immediate postpartum testing (6-12 weeks) without delay and intensified annual surveillance (e.g., every 6-12 months) rather than the standard 1-3 year intervals. These recommendations are extrapolated from the high risk magnitude (OR > 10) observed. Women meeting the criteria for overt diabetes should be considered to have established high-risk metabolic disease until proven otherwise. The window of opportunity for prevention in this group may be much narrower than that for women with standard GDM, requiring immediate postpartum transition to diabetes prevention programs and intensive cardiovascular risk factor modification.

Future clinical trials for postpartum diabetes prevention should specifically stratify randomization by overt diabetes versus standard GDM status. The overt diabetes phenotype, likely driven by severe β-cell dysfunction, represents a high event rate cohort that may require distinct pharmacologic interventions (e.g. early metformin or incretin therapies) rather than lifestyle modification alone.

Strengths

The strengths of this review include the rigorous application of the NOS [12] and JBI [13] appraisal tools, which confirmed a low risk of bias among the included studies. The use of the HKSJ adjustment in our random-effects models ensured robust error estimation despite the modest number of studies. Furthermore, the high certainty of evidence (GRADE) [25] for our primary outcome provides confidence in the validity of the estimate.

Limitations

Most data were derived from observational cohorts, precluding definitive causal inferences. There was variability in the timing of postpartum follow-up, ranging from six weeks to six years, although meta-regression did not identify follow-up duration as a significant factor. Additionally, while significant risks were identified for surrogate cardiovascular markers and metabolic syndrome, there is a paucity of long-term data on hard cardiovascular endpoints (e.g. myocardial infarction and stroke) specifically comparing overt diabetes to GDM, highlighting a critical gap for future longitudinal research. While the association with T2DM is robust, conclusions regarding subclinical cardiovascular pathology (e.g. BMI changes) remain tentative due to data heterogeneity. As most data derive from tertiary centers, these findings may reflect a higher-risk subset of the general population, potentially limiting generalizability to community settings.

Subgroup and meta-regression analyses should be interpreted as exploratory given the limited number of studies (k<10). Furthermore, while we utilized adjusted estimates, residual confounding from unmeasured factors such as postpartum lifestyle changes and breastfeeding duration cannot be ruled out. 

## Conclusions

Women diagnosed with overt diabetes during pregnancy represent a vulnerable population with a 10-fold higher risk of early postpartum diabetes and a doubled risk of metabolic syndrome compared to women with GDM. These findings challenge the current one-size-fits-all approach to postpartum care. Clinical guidelines should be updated to recognize overt diabetes as a distinct, high-priority indicator of intensive, lifelong cardiometabolic surveillance and risk reduction.
